# Increased circulating cell signalling phosphoproteins in sera are useful for the detection of pancreatic cancer

**DOI:** 10.1038/sj.bjc.6605734

**Published:** 2010-06-15

**Authors:** S Takano, K Sogawa, H Yoshitomi, T Shida, K Mogushi, F Kimura, H Shimizu, H Yoshidome, M Ohtsuka, A Kato, T Ishihara, H Tanaka, O Yokosuka, F Nomura, M Miyazaki

**Affiliations:** 1 Department of General Surgery, Graduate School of Medicine, Chiba University, 1-8-1 Inohana, Chuo-ku, Chiba 260-8670, Japan; 2 Department of Molecular Diagnosis, Graduate School of Medicine, Chiba University, Chiba, Japan; 3 Clinical Proteomics Research Center, Chiba University Hospital, Chiba, Japan; 4 Information Center for Medical Sciences, Tokyo Dental and Medical University, Tokyo, Japan; 5 Department of Medicine and Clinical Oncology, Graduate School of Medicine, Chiba University, Chiba, Japan

**Keywords:** serum marker, pancreatic cancer, phospho-extracellular signal-regulated kinase, early diagnosis, cell signalling

## Abstract

**Background:**

Intracellular phosphoprotein activation significantly regulates cancer progression. However, the significance of circulating phosphoproteins in the blood remains unknown. We investigated the serum phosphoprotein profile involved in pancreatic cancer (PaCa) by a novel approach that comprehensively measured serum phosphoproteins levels, and clinically applied this method to the detection of PaCa.

**Methods:**

We analysed the serum phosphoproteins that comprised cancer cellular signal pathways by comparing sera from PaCa patients and benign controls including healthy volunteers (HVs) and pancreatitis patients.

**Results:**

Hierarchical clustering analysis between PaCa patients and HVs revealed differential pathway-specific profiles. In particular, the components of the extracellular signal-regulated kinase (ERK) signalling pathway were significantly increased in the sera of PaCa patients compared with HVs. The positive rate of p-ERK1/2 (82%) was found to be superior to that of CA19-9 (53%) for early stage PaCa. For the combination of these serum levels, the area under the receiver-operator characteristics curves was showing significant ability to distinguish between the two populations in independent validation set, and between cancer and non-cancer populations in another validation set.

**Conclusion:**

The comprehensive measurement of serum cell signal phosphoproteins is useful for the detection of PaCa. Further investigations will lead to the implementation of tailor-made molecular-targeted therapeutics.

Pancreatic cancer (PaCa) is an exceptionally devastating disease with a 5-year survival rate of only 5% (Jemal *et al*, 2009). One of the most crucial reasons for the poor prognosis is the lack of early diagnostic markers for PaCa. To overcome pancreatic malignancy, there is an urgent need to discover highly sensitive markers for early detection. The widely used serum-circulating marker for PaCa, carbohydrate tumour-associated antigen 19-9 (CA19-9), is not sufficiently accurate to be used as a diagnostic marker. CA19-9 is elevated in only approximately 65% of individuals with a resectable PaCa, and is also frequently elevated in patients with various benign pancreaticobiliary disorders; notably cholestasis and chronic pancreatitis (PT) (Goggins, 2005). Thus, CA19-9 is not recommended for diagnostic purposes (Locker *et al*, 2006).

In the past decade, various approaches have been used to discover new cancer serum biomarkers, and have identified some attractive molecular targets as diagnostic or prognostic markers for PaCa (Gold *et al*, 2006; Takano *et al*, 2008). Despite the identification of candidate proteins that have high diagnostic sensitivity and specificity in validation tests, translating these research findings to useful and reliable clinical tests still remains difficult (Zhang *et al*, 2004; Petricoin *et al*, 2002).

Protein phosphorylation is one of the most prominent, and intensively studied post-translational modifications in biological systems. Specifically, better understanding of the defective or hyperactive signalling pathways in cancer cells has been the major focus of mechanistic studies of cancer progression and differentiation, as well as in the identification of candidate markers for diagnosis and therapeutic targets (Petricoin *et al*, 2005). Ultimately, the signalling pathways promote tumourigenesis through the coordinated phosphorylation of proteins that directly regulate protein synthesis, cell-cycle progression and of transcription factors that regulate the expression of genes involved in these processes. Although these intracellular signalling pathways and its components (activated or inactivated forms) that are closely associated with cancer progression are among the most thoroughly studied in molecular cancer research, there has been little understanding of the dynamic nature of these circulating proteins in the bloodstream.

Because blood continuously perfuse the tissues of the body, it is thought to contain most human proteins (at least in fragment forms), thereby supplying the richest and most detailed source of information about the physiological state of the body (Anderson *et al*, 2004). Thus, blood has a pivotal role in early disease detection. The turnover of proteins in cells requires calculated degradation processes to secrete intact or fragment forms into blood and to also remove proteins that are no longer necessary or those that have lost functional capabilities. Meanwhile, it is thought that a large part of those human serum-circulating proteins that promote signal transduction in cells may be cleaved by degradation of endogenous substrates and proteases (Villanueva *et al*, 2006a, 2006b; Schilling and Knapp, 2008). Taken together, it is worthwhile for the early cancer detection to elucidate the molecular networks in cancerous cells and the microenvironments, and to investigate the dynamic nature of those cancer-related phosphorylated proteins including the fragments in circulating blood.

The Bio-Plex suspension array using specific antibodies and based on the principle of flow cytometry is a high-throughput technology that can measure multiple proteins in low sample volumes. Immunoaffinity approaches, particularly phospho-specific antibodies that recognise low abundance phospho-tyrosine, -serine and -threonine residues in the specific epitopes, have been used to assess phosphoprotein enrichment. In further developing this technology, it is possible to detect the phospho-specific sites of the parent molecule and its degraded fragments in serum as early diagnostic markers by comprehensive analysis in cancer patients. This technology has overcome a critical problem for the translational application of proteomics by developing a procedure that is convenient with high sensitivity, specificity and reproducibility. Moreover, this may also provide important information regarding the activation state of kinase-driven signalling networks in each cancer patient for therapeutic target selection with the advantage that the screen is less invasive.

In this study, we analysed the circulating cell signalling phosphoproteins in sera using this proteomic approach and investigated whether these protein levels are useful for detection, as early diagnostic serum markers for PaCa, in combination with comprehensive and hierarchical cluster analyses. Our study results indicate that the use of this new approach will lead to new insights in proteomic cancer biology and in the potential development of patient-tailored combination molecular targeting therapy, through elucidation of the phosphoprotein networks in serum from cancer patients.

## Materials and methods

### Patient samples

We selected four populations of patients with PaCa, PT and healthy control volunteer (HV). Blood samples were obtained from all patients who were diagnosed with PaCa and PT in the Chiba University Hospital, Chiba, Japan, from November 2002 to March 2009, and the samples were also obtained from HVs in the Chiba University Hospital and the Kashiwado Hospital, Chiba, Japan. Sera were collected from 26 patients with PaCa and 25 HVs for the training set, from 80 patients with PaCa and 68 HVs for validation set 1, furthermore, to assess the diagnostic ability of discriminating between cancer and non-cancer populations, sera of 35 patients with PaCa as a cancer group, and 40 patients with PT as well as 48 HVs as a non-cancer group were selected to match for age for validation set 2 (Table 1). All patients were histologically confirmed as PaCa. The characteristics of 141 patients with PaCa are summarised in Table 2. All blood samples were processed according to a standardised protocol, and serum sample aliquots were frozen until the subsequent analysis. None of the patients received any therapeutic treatments, such as radiation, chemotherapy or operation, until serum samples were collected. The ethics committee for each institute approved the protocol. Written informed consent was obtained from all patients and HVs.

### Bio-Plex phosphoprotein suspension assay

Phosphorylated proteins in serum were detected with a Bio-Rad phosphoprotein immunoassay kit using the Bio-Plex 200 suspension array system (Bio-Rad Laboratories, Hercules, CA, USA). The human serum diluent buffer was added up to 50 *μ*l to the eight-fold diluted samples, 50 *μ*l aliquots of each of the diluted samples were plated in the 96-well filter plate, coated with anti-phosphoprotein antibody-coupled beads, and incubated for 16 h on a platform shaker at 300 r.p.m. at room temperature. The wells were vacuum filtered and washed, 1 *μ*l of detection antibodies (25 × ) was added, vortexed and incubated for 30 min. After additional vacuum filtration and washing of the wells, 0.5 *μ*l streptavidin–PE (100 × ) was added to each well and allowed to incubate for 10 min. The wells were again vacuum filtered and washed, 125 *μ*l of re-suspension buffer was added and incubated for 30 s. Data acquisition and analysis were performed using Bio-Plex Manager software version 5.0. The data of measurement by the Bio-Plex 200 suspension array system are presented in the Supplementary Information (Supplementary Figure S1; Supplementary Table S1).

For the training set, 18 targeted phosphorylated (p-) proteins were measured using Bio-Plex 200 suspension array system in the comprehensive phosphoprotein analysis. Focusing on the more promising candidate proteins, phospho-mitogen-regulated kinase 1 (p-MEK1), phospho-extracellular signal-regulated kinases 1/2 (p-ERK1/2) and those total proteins; we measured (*t*-) for the further validation sets.

### Immunohistochemistry

Paraffin-embedded tissues were cut into 4 *μ*m-thick serial sections and were de-paraffinised. Serial section slides were placed in citric acid buffer (10 mmol l^−1^, pH 6.0) with 0.2% Tween 20 and boiled in a microwave oven (2 × 6 min) to retrieve the antigen. The slides were then rinsed and blocked in a 3% H_2_O_2_ solution with methanol for 10 min, before being incubated overnight at 4°C with the primary antibodies; rabbit anti-phospho-MEK1/2 monoclonal antibody (Cell Signaling Technology, Beverly, MA, USA) and rabbit anti-phospho-ERK1/2 (p44/42 MAPK) monoclonal antibody (Cell Signaling Technology) (1 : 50 and 1 : 200 dilution respectively). SignalStain Antibody Diluent (Cell Signaling Technology) was used as the dilution buffer. They were then rinsed in PBS, and incubated for 60 min with secondary antibody labelled with streptoavidin–biotin–peroxidase (DAKO LSAB+ kit; DakoCytomation, Glostrup, Denmark). The bound complex was visualised using diaminobenzidine liquid chromogen and counterstained with hematoxylin.

### Comprehensive and hierarchical clustering analyses of the training and validation sets

To investigate the similarity of expression patterns, we performed hierarchical clustering analysis using R statistical software (version 2.8.0). Before analysis, the expression levels were standardised using *Z*-transformation (mean=0 and variance=1) for each protein. We then used Euclidian distance of expression patterns for calculation of distance matrices (i.e., one for proteins and the other for samples) between each variable, as well as the average linkage method for clustering analysis.

### Multivariate logistic regression using selected proteins

To assess the diagnostic ability for PaCa patients and controls, we performed univariate and multivariate logistic regression analyses using p-ERK1/2, CA19-9 and the combination of p-ERK1/2 and CA19-9 models. Receiver-operating characteristic (ROC) curves and area under the curve (AUC) based on the prediction results of the obtained regression models were calculated by the R statistical software.

### Statistical analysis

Statistical analyses were performed using the appropriate tests as indicated. *P*-values <0.05 were considered statistically significant. To compare the positive rate for detecting early stage pancreatic malignancies, we determined the positive levels of p-ERK1/2 in disease patients by the reference values in each of the respective three sets (Solberg, 1987). The reference values in the three sets were calculated using reference limits corresponding to 0.95 fraction of the distribution, that is the upper limit of the 95% confidence interval (CI), in the three respective healthy control groups. For CA19-9, a cut-off value of 37 IU ml^−1^ was used, according to the manufacturer's specifications for the reference range of CA19-9.

## Results

### Circulating phosphoproteins levels are increased in sera from patients with pancreatic cancer

**Figure 1:**
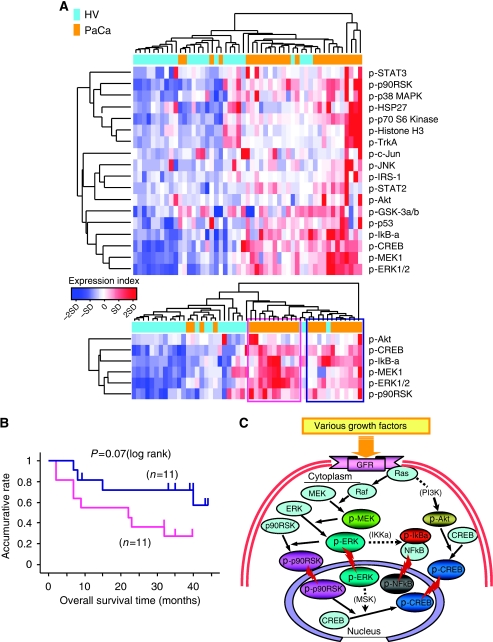
Hierarchical clustering of phosphoprotein expression profiles in the training set. (**A**) Healthy volunteers (HVs) are denoted in light blue in the vertical side bar, and pancreatic cancer (PaCa) patients are denoted in orange. Dendrograms show the classification determined by hierarchical clustering analysis of 18 targeted phosphoproteins. As shown in Expression index, red and blue in the matrix indicate relative overexpression and underexpression respectively (s.d.; standard deviation). The hierarchical clustering analysis clearly distinguished the two groups; the majority of PaCa patients are found in the right side, whereas HVs are mainly located in the left side of the heat map (upper panel). The profiles of six candidate circulating phosphoproteins associated with PaCa are revealed by hierarchical clustering analysis (lower panel). Subclass analysis separated the PaCa patients into two groups based on the candidate phosphoproteins. The distinction of the two groups is indicated by the blue and purple lined boxes. (**B**) Kaplan–Meier analysis revealed that each of the two groups (blue and purple lines) distinguished by the hierarchical clustering analysis was well matched with a favourable and unfavourable prognosis of PaCa patient groups. (**C**) The schema of the correlation between candidate phosphoproteins by cell signal transduction in the intracellular environment. The crucial interaction among these molecules allows the cancer to proliferate and differentiate through representative cell signalling pathways.

To detect new biomarkers characteristic of the PaCa patients, we comprehensively first measured the 18 major targeted cell signalling phosphoproteins levels in sera of the training set using the Bio-Plex suspension array. Many of the target phosphoproteins levels were increased significantly in sera from PaCa patients compared with the HVs (see detail of the experimental data in Supplementary Table S2). Hierarchical clustering analysis showed that the relative differential expressions of circulating phosphoproteins clearly distinguished PaCa patients from HVs (Figure 1A). Six candidate phosphoproteins (p-ERK1/2: *P*<0.00001, p-MEK1: *P*<0.0005, phospho-p90 ribosomal S6 kinase (p-p90RSK): *P*<0.0001, phospho-cAMP response element binding protein (p-CREB): *P*<0.00001, p-Akt: *P*<0.00005 and p-I*κ*B-*α*: *P*<0.0001; Mann–Whitney *U*-test) were significantly increased in sera from patients with PaCa compared with the HVs.

As shown in the lower panel of Figure 1A, similar cluster structures were obtained in the clustering analysis of these six phosphoproteins. Subclass analysis separated the PaCa patients into two groups, based on hierarchical clustering of the six candidate marker levels, and revealed that each of the two groups correlated well with the groups that had favourable and unfavourable prognoses (*P*=0.07; log-rank test; Figure 1B). These were also closely correlated with each protein belonging to the phosphatidylinositol-3-OH kinase/Akt, NF-*κ*B and ERK signalling pathways that are crucial for cancer survival (Figure 1C).

**Figure 2:**
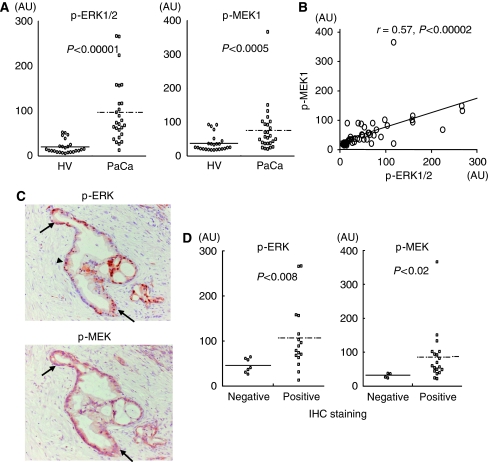
Serum phosphoproteins increased in PaCa patients compared to HVs in the training set. (**A**) Circulating p-ERK1/2 and p-MEK1 levels in sera are significantly greater in PaCa patients than HVs. (**B**) Linear regression with p-ERK1/2 and p-MEK1 levels reveals a significant, positive correlation in the training set. (**C**) Immunostaining for p-ERK and p-MEK in PaCa tissues (original magnification, × 200). Note that expression of the two phosphoproteins is evident in the cancerous cytoplasm (arrows in p-ERK and p-MEK) and nucleus (arrowhead in p-ERK), and is also found in stromal cells surrounding the ductal carcinoma cells. (**D**) Both p-ERK and p-MEK levels in sera were significantly correlated with the positive staining of PaCa tissues (p-ERK: *P*<0.008, p-MEK: *P*<0.02; Mann–Whitney *U*-test).

Two of these proteins, p-ERK1/2 and p-MEK1, are shown in Figure 2A. Of particular interest, four of the six phosphoproteins were proteins directly associated with the most popular pathway of pancreatic carcinogenesis, the Ras/Raf/MEK/ERK signalling cascade to two proteins (p-p90RSK and p-CREB) that are directly or indirectly phosphorylated by ERK. Surprisingly, the results indicate that p-ERK1/2 levels in serum showed a significantly positive correlation with p-MEK1 levels (*r*=0.57, *P*<0.00002; Pearson's correlation coefficient test) as well as p-ERK, which would theoretically be dependant on the activity of MEK and is in turn promoted by an entire series of upstream events (Figure 2B). Therefore, we mainly selected two key molecules, ERK and MEK, to investigate the expression of those phosphorylated and total proteins in sera for further validation analyses by Bio-Plex assay.

### Confirmation of target phosphoprotein serum levels by western blot analysis

To confirm the results obtained from Bio-Plex assay, we assessed the expression of phosphoproteins both sera from patients with PaCa and HVs by immunoprecipitation assay and western blot analysis. Corresponding with Bio-Plex data, increased p-ERK1/2 expression levels were confirmed by western blot analysis in sera from three PaCa patients and one HV (see detail of the methods and experimental data in Supplementary Figure S2).

### Activated p-ERK and p-MEK are expressed in pancreatic cancer cells

To examine the potential source of the activated ERK and MEK in serum, we performed immunohistochemical staining for these phosphoproteins in resected PaCa tissues. As shown in Figure 2C, both activated p-ERK and p-MEK expression levels were clearly positive in pancreatic ductal carcinoma cells. The p-ERK and p-MEK expression was localised to the neoplastic epithelial cells and some stromal cells especially surrounding the ductal carcinoma cells. Activated p-ERK was found in both the nucleus and cytoplasm of cancer cells. Conversely, p-MEK expression was not found in the nucleus of cancer cells, but stained intensely in the cytoplasm of cancer cells.

### Both p-ERK and p-MEK levels in sera were significantly correlated with the positive staining of pancreatic cancer tissues

Next, we investigated whether the two phosphoproteins levels in sera correlated with the positive staining in PaCa tissues. Among 26 PaCa patients in training set, 23 patients (R0, R1 and R2; all resectable cases) were analysed by immunohistochemical staining of the PaCa tissues. The 23 patients were divided into two groups based on positive or negative staining in PaCa cells, 16 (69.6%) of 23 cases were p-ERK-positive staining, and 19 (82.6%) cases were p-MEK-positive staining. Notably, as shown in Figure 2D, both p-ERK and p-MEK levels in sera were significantly correlated with the positive staining of PaCa tissues respectively (p-ERK: *P*<0.008, p-MEK: *P*<0.02; Mann–Whitney *U*-test).

### Both phospho- and total-ERK1/2 simultaneously increase with a positive correlation in sera of patients with pancreatic cancer

**Figure 3:**
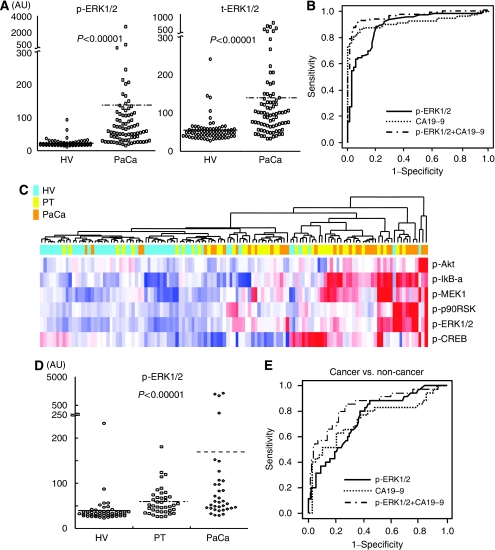
Confirmation of the results obtained from the training set for validation set 1. (**A**) The similar result of the training set shows that both circulating p-ERK1/2 and t-ERK1/2 levels were significantly increased in sera from PaCa patients compared with that of HVs for validation set 1. (**B**) The ROC analyses were performed for the serum levels of p-ERK1/2, CA19-9 and a combination of p-ERK1/2 and CA19-9 between PaCa patients and HVs. The respective AUCs were 0.88 for p-ERK1/2 level, 0.91 for CA19-9 level and 0.96 for the combination of p-ERK1/2 and CA19-9 levels. Comparing among three populations in validation set 2. (**C**) Serum levels of six candidate phosphoproteins were able to distinguish among the three populations (HV, PT and PaCa) by hierarchical clustering analysis. The analysis distinguished the three groups; the majority of PaCa patients are found on the right side, whereas PT are located diffusely in the approximate centre and HVs are mainly located on the left side of the heat map. (**D**) Circulating p-ERK1/2 levels in sera were significantly differenced among three populations (PaCa, PT and HVs) (*P*<0.00001; Kruskal–Wallis test). (**E**) The ROC analyses were performed for the serum levels of p-ERK1/2 and CA19-9 between cancer (PaCa) and non-cancer (HVs and PT) populations. The respective AUCs were 0.75 for p-ERK1/2 level and 0.70 for CA19-9 level and 0.84 for the combination of p-ERK1/2 and CA19-9 levels.

To confirm the results obtained from the training set, we measured and analysed both ERK and MEK serum levels with an increased sample size in validation set 1. Similar results were obtained, that both p-ERK1/2 and p-MEK1 levels were significantly increased in sera from PaCa patients compared with that of HVs for validation set 1 (p-ERK1/2; *P*<0.00001, p-MEK1; *P*<0.00001: Mann–Whitney *U*-test; Figure 3A). In addition, t-ERK1/2 levels were also significantly more upregulated in sera from PaCa patients compared with that of HVs in validation set 1 (*P*<0.00001; Mann–Whitney *U*-test). Of particular interest, both p- and t-ERK1/2 levels increased simultaneously with a positive correlation in sera from PaCa patients (*r*=0.38, *P*<0.0004; Pearson's correlation coefficient test) (figure not shown).

### Phospho-ERK1/2 level in serum excels in the detection of pancreatic cancer

To estimate the cell signalling phosphoprotein, p-ERK1/2, as a novel serum biomarker to detect PaCa patients, we calculated the ROC curves, which correlate the true- and false-positive rates (sensitivity and 1 specificity) between PaCa patients and HVs. The area under the ROC curve (AUC) was 0.94 for p-ERK1/2, and concerning with other phosphoproteins, the AUCs were 0.79 for p-MEK1, 0.81 for p-p90RSK, 0.86 for p-CREB and 0.83 for p-Akt in the training set. To validate and compare the abilities of serum markers for the diagnosis of PaCa, we constructed ROC curves for p-ERK1/2, CA19-9 and the combination of two serum levels in validation set 1. The respective AUC was 0.88 for p-ERK1/2, 0.91 for CA19-9 and 0.96 for the combination p-ERK1/2 and CA19-9 (Figure 3B).

The positive rate of serum p-ERK1/2 in the disease groups was calculated using the reference values determined according to the upper limit of 95% CI in the three respective healthy control groups. In all three sets of this study, only five patients showed negative levels for both p-ERK1/2 and CA19-9. For CA19-9, 39 false-negative patients were mostly picked up as p-ERK1/2-positive (87.2%) patients with PaCa (Table 3). These results indicate that the combination of p-ERK1/2 and CA19-9 achieved sufficiently high sensitivity and specificity to diagnose PaCa accurately by supplementing the low sensitivity of CA19-9 that was caused by deficiency of Lewis antigens and so on.

### Combination of p-ERK1/2 and CA19-9 levels is superior discriminatory power between cancer and non-cancer populations

In validation set 2, we compared and analysed the differential protein expression of six candidate phosphoproteins levels in sera from PaCa, PT patients and HVs. Hierarchical clustering analysis indicated that the populations of PT patients were located diffusely but approximately in between the HVs and PaCa patient groups (Figure 3C).

Furthermore, to assess the discriminatory power of serum p-ERK1/2 levels, we measured to compare p-ERK1/2 and CA19-9 levels in sera of PaCa patients and age-matched benign controls including PT patients. Phospho-ERK1/2 levels were significantly increased in sera among three populations (*P*<0.00001; Kruskal–Wallis test; Figure 3D), and between cancer and non-cancer populations (*P*<0.00002; Mann–Whitney *U*-test). To discriminate cancer from non-cancer groups, we performed multivariate logistic regression analysis using p-ERK1/2 and CA19-9. As a result, both p-ERK1/2 (odds ratio: 13.4, 95% CI: 2.14–83.6, *P*=0.0056; Wald test) and CA19-9 (odds ratio: 3.67, 95% CI: 1.86–7.22, *P*=0.0002; Wald test) were identified as significant variables for the detection of PaCa. For distinguishing between cancer and non-cancer groups, the respective AUC was 0.75 for p-ERK1/2 and 0.70 for CA19-9, and the AUC was 0.84, showing high ability to distinguish between cancer and non-cancer groups, for the combination of the two serum levels (Figure 3E). These results suggest the combination of p-ERK1/2 and CA19-9 levels is better discriminatory power compare to CA19-9 alone between cancer and non-cancer populations.

### Circulating p-ERK1/2 is a potential novel marker for early stage of pancreatic cancer

To emphasise the diagnosis of early stage of patients with pancreatic malignancy, we found that the sensitivity of serum p-ERK1/2 levels for predicting stage I PaCa in our study population was 82% (14 out of 17 patients with stage IA or IB cancers had elevated p-ERK1/2), whereas only 9 out of 17 (53%) patients showed elevated CA19-9. These results suggest that the measurement of serum p-ERK1/2 levels could be particularly helpful in the detection of early stage PaCa.

## Discussion

The results reported herein show that the measurement of circulating signal transduction proteins in serum led to the detection of PaCa. To elucidate molecules related to PaCa progression, we used a new strategy based on the multiplexed cell signalling of phosphoproteins in serum by hierarchical clustering analysis. To detect pre-malignant tumour or early stage malignancies, it is necessary to be able to assess very low abundant substances that are likely produced by tumour itself (i.e., fragments of cellular components, endo- or exogenous protease and secretion derived from tumour) (Villanueva *et al*, 2006a, 2006b), the microenvironment of the tumour–host interface (Iacobuzio-Donahue *et al*, 2002) and the host immune response to tumour (Koomen *et al*, 2005). Recently, it was reported that both lymphatic vessel compression with resultant functional abnormalities and elevated interstitial fluid pressure occur during the early stages of carcinogenesis (Hagendoorn *et al*, 2006). These insights have formed the theoretical foundation for the detection of early stages of cancer.

Biological fluids, such as serum, are a readily obtainable source of potential cancer biomarkers that are shed or secreted by cancer cells, and are produced as a consequence of humoral immunity (Lu *et al*, 2008). Serum immerses most tissues in the body and is therefore likely to contain cell-derived proteins that can provide dynamic information about various biological processes. In addition, it is thought that cellular or tissue protein might likely present as a full-length form or the cleavage fragments that freely enter circulation by diffusion or convection (Liotta and Petricoin, 2006).

Concerning proteins as indicators, it has been recognised that blood protein biomarkers are amplified in the circulatory system because they accumulate on the high concentration of resident proteins, such as albumin, and then acquire the longer half-life of albumin, thereby protecting the bound species from renal clearance (Lowenthal *et al*, 2005; Araujo *et al*, 2008). Lowenthal *et al* also indicated that among many individual sequences that were predicted from albumin-associated proteins in serum from patients with three stages of ovarian cancer, the predicted sequences were largely fragments derived from proteins with diverse biological functions, including crucial cellular signal transduction factors. Interestingly, the kinds of signal transduction factors were more numerous in sera from patients with early stage than in advanced stage of cancer among the identified proteins. In an recent study, the enrichment of serum phosphopeptides using the modified particles was successful to identify phosphorylated peptides that were related to cancer. The profiling of these degraded fragments has been found to be able to distinguishing between hepatocellular carcinoma patients and healthy individuals (Hu *et al*, 2009). Our current study results are consistent with these theories of protein amplification and actual identification in the circulatory system.

The activation of epidermal growth factor receptor (EGFR) and the various downstream targets, such as Ras, Raf, MEK and ERK, are deeply implicated in the pathogenesis of PaCa with malignant transformation and enhanced tumour aggressiveness. In addition, the signalling cascade is likely crucial for PaCa progression because K-Ras gene mutations have been found in many populations of human PaCa specimens. The efficacy of molecular targeting therapies for PaCa, such as an inhibitor of EGFR tyrosine kinase, small-molecule inhibitor of Raf kinase and that of the dual specificity kinase MEK1/2, have recently being evaluated in some clinical trials, however, the results have not been impressive (Rinehart *et al*, 2004; Siu *et al*, 2006; Moore *et al*, 2007). The major reason is considered that the dysregulation or hyperactivity in the network of intracellular and extracellular signalling pathways is so complicated with multiplicity that each individual may have a differential profile even among similar malignancies. It is reasonable to surmise that to obtain maximum efficacy of molecular-targeted therapies it is necessary to investigate which pathway is more highly activated for each cancer patient (Jimeno *et al*, 2008). In the near future, our new insights may resolve this problem with a minimally invasive approach.

This is the first study to show circulating cell signalling phosphoproteins in blood of PaCa patients. In our experiments, comprehensive and hierarchical clustering analyses of serum phosphoproteins between PaCa patients and HVs revealed pathway-specific profiles, in particular components of the ERK signalling pathway, and a new method to classify serum phosphoproteins possibly derived from tumour itself, based on intracellular signalling portraits. As mentioned above, overcoming the issue of specificity as well as discovering highly sensitive markers for early detection are undoubtedly important. We confirmed that this signature could be used to discriminate not only between cancer and healthy controls in an independent validation set but also between cancer and non-cancer populations in an age-matched sample as another validation set. We also found that these circulating molecules were potentially useful for the diagnosis of early stage PaCa. These results suggest that the level of circulating p-ERK may be associated with early stage of pancreas carcinogenesis.

Immunohistochemistry of PaCa tissues showed that two target phosphoproteins, p-ERK and p-MEK, were simultaneously well expressed during the early stage neoplasms, even in the cancer cells of non-invasive or minimally invasive ductal carcinoma, as well as in the advanced stage patients with PaCa. Furthermore, both p-ERK and p-MEK levels in sera of PaCa patients were in good correlation with the positive staining of their PaCa tissues. Taken together, we consider that the major source for the elevation of cell signal phosphoproteins levels in serum may be cancer cells that are showing augmented cell signalling.

Subclasses distinguished by hierarchical clustering analysis of six candidate markers indicated good correlation with the prediction of the prognosis of PaCa patients in this study. Further investigation of subclass analysis by hierarchical clustering will provide fruitful information regarding which factors of cell signalling phosphoproteins in serum are associated with the malignant behaviour of PaCa.

PaCa develops as a result of the stimulation and activation of various growth factor receptors. The continuous stimulation of these signal transduction pathways leads to increases in both the activated and inactivated forms of the cell signalling molecules in the intracellular environments of cancerous cells. The downstream activation transmits information through post-translational protein modifications with reversible protein phosphorylation. Increasing signal molecules that accumulate in the cell trigger changes in the penetration of cell membrane, which causes the release of both phosphoproteins and the degraded fragments to extracellular environments by cellular apoptosis. Once released from the intracellular environments, those proteins likely lose their original function, and are then carried to nearby blood vessels, and circulate freely or with binding to high-affinity transfer proteins in blood circulatory system. However, further research is needed to elucidate the sequence of this pathway.

In conclusion, we found cancer-associated cell signal phosphoproteins in serum using multiplexed cell signalling analysis. The measurement of circulating phosphoproteins in serum was able to discriminate between cancer patients and benign controls, and this new approach was helpful in the early diagnosis of patients with PaCa. This method shows the feasibility of this analysis, with a less invasive approach. The next step is to elucidate the profiling of cell signal activation by these comprehensive and hierarchical clustering analyses may discriminate subclasses into clinically significant groups. In the near future, investigations determining the footprints of circulating phosphoproteins will lead to the clinical application of this method that will be used for targeted tailor-made therapeutics.

## Figures and Tables

**Table 1:** Summary of all participants

**Experimental groups (number of patients)**	**Sex (M/F)**	**Age (mean±s.d.)**
*Training set (n*=*51)*		
Pancreatic cancer (*n*=26)	17/9	65.2±8.0
Healthy volunteer (*n*=25)	17/8	52.0±10.6
		
*Validation set 1 (n*=*148)*		
Pancreatic cancer (*n*=80)	48/32	62.9±10.8
Healthy volunteer (*n*=68)	41/27	54.1±6.8
		
*Validation set 2 (n*=*123)*		
Pancreatic cancer (*n*=35)	21/14	63.6±8.8
Pancreatitis (*n*=40)	38/2	61.7±8.8
Healthy volunteer (*n*=48)	32/16	62.4±7.3

Abbreviations: F=female; M=male.

**Table 2:** Characteristics of patients with pancreatic cancer

**Variables**	**Training set (*n*=26)**	**Validation set 1 (*n*=80)**	**Validation set 2 (*n*=35)**
*Tumor stage*			
T1	0	3	8
T2	2	2	4
T3	21	47	12
T4	2	10	2
TX	1	18	9
			
*Nodal status*			
N0	7	16	16
N1	17	41	10
NX	2	23	9
			
*Metastasis*			
M0	24	53	30
M1	2	27	5
			
*UICC stage*			
IA	0	2	8
IB	1	2	4
IIA	6	11	3
IIB	16	32	10
III	1	6	5
IV	2	27	5
			
*Resection status*			
R0	12	38	21
R1	7	9	5
R2	4	6	0
RX	3	27	9

Abbreviations: RX=unresectable case; UICC=Union Internationale Contre le Cancer.

**Table 3:** p-ERK1/2-positive rate in CA19-9 false-negative patients with pancreatic cancer

	**CA19-9 false negative (%)**	**p-ERK1/2-positive in CA19-9 false negative (%)**
Training set	6/26 (23.1)	6/6 (100.0)
Validation set 1	22/80 (27.5)	20/22 (90.9)
Validation set 2	11/35 (31.4)	8/11 (72.7)
Total	39/141 (27.7)	34/39 (87.2)

Abbreviations: CA19-9=carbohydrate tumour-associated antigen 19-9; p-ERK1/2=phospho-extracellular signal-regulated kinases 1/2.
