# Supplementary material for: Increased circulating cell signalling phosphoproteins in sera are useful for the detection of pancreatic cancer
Source: Br J Cancer. 2010 Jun 15;103(2):223–31. doi: 10.1038/sj.bjc.6605734 (PMC2906731; doi:10.1038/sj.bjc.6605734)
Supplement: Supplementary Figure S1 [file 6605734x1.ppt]

## Slide 1
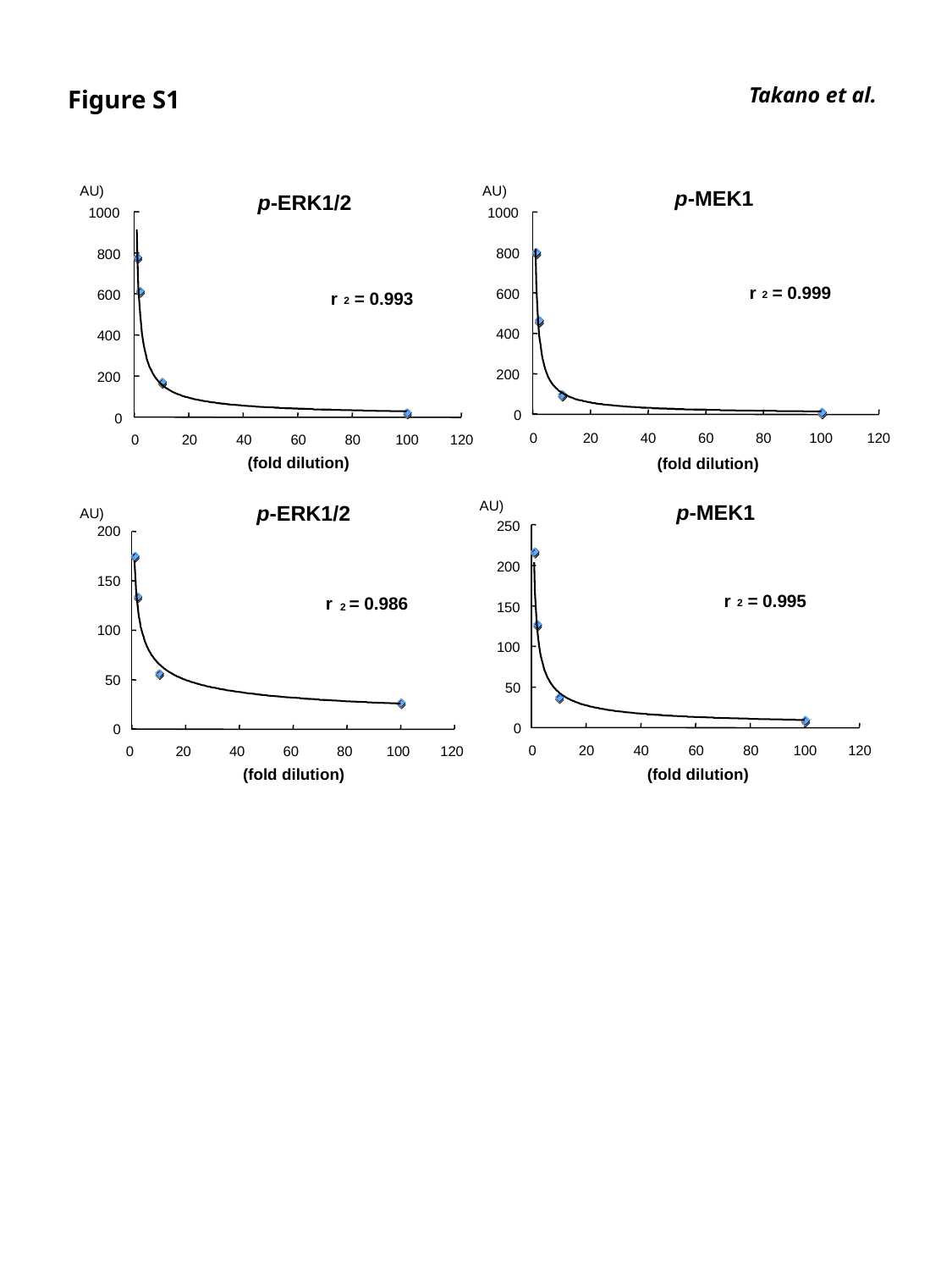

Takano et al.
Figure S1
AU)
AU)
p-MEK1
p-ERK1/2
1000
1000
800
800
r
 = 0.999
600
600
2
r
 = 0.993
2
400
400
200
200
0
0
0
20
40
60
80
100
120
0
20
40
60
80
100
120
(fold dilution)
(fold dilution)
AU)
p-MEK1
p-ERK1/2
AU)
250
200
200
150
r
 = 0.995
r
 = 0.986
2
2
150
100
100
50
50
0
0
0
20
40
60
80
100
120
0
20
40
60
80
100
120
(fold dilution)
(fold dilution)
