# Supplementary material for: Increased circulating cell signalling phosphoproteins in sera are useful for the detection of pancreatic cancer
Source: Br J Cancer. 2010 Jun 15;103(2):223–31. doi: 10.1038/sj.bjc.6605734 (PMC2906731; doi:10.1038/sj.bjc.6605734)
Supplement: Supplementary Figure S2 [file 6605734x2.ppt]

## Slide 1
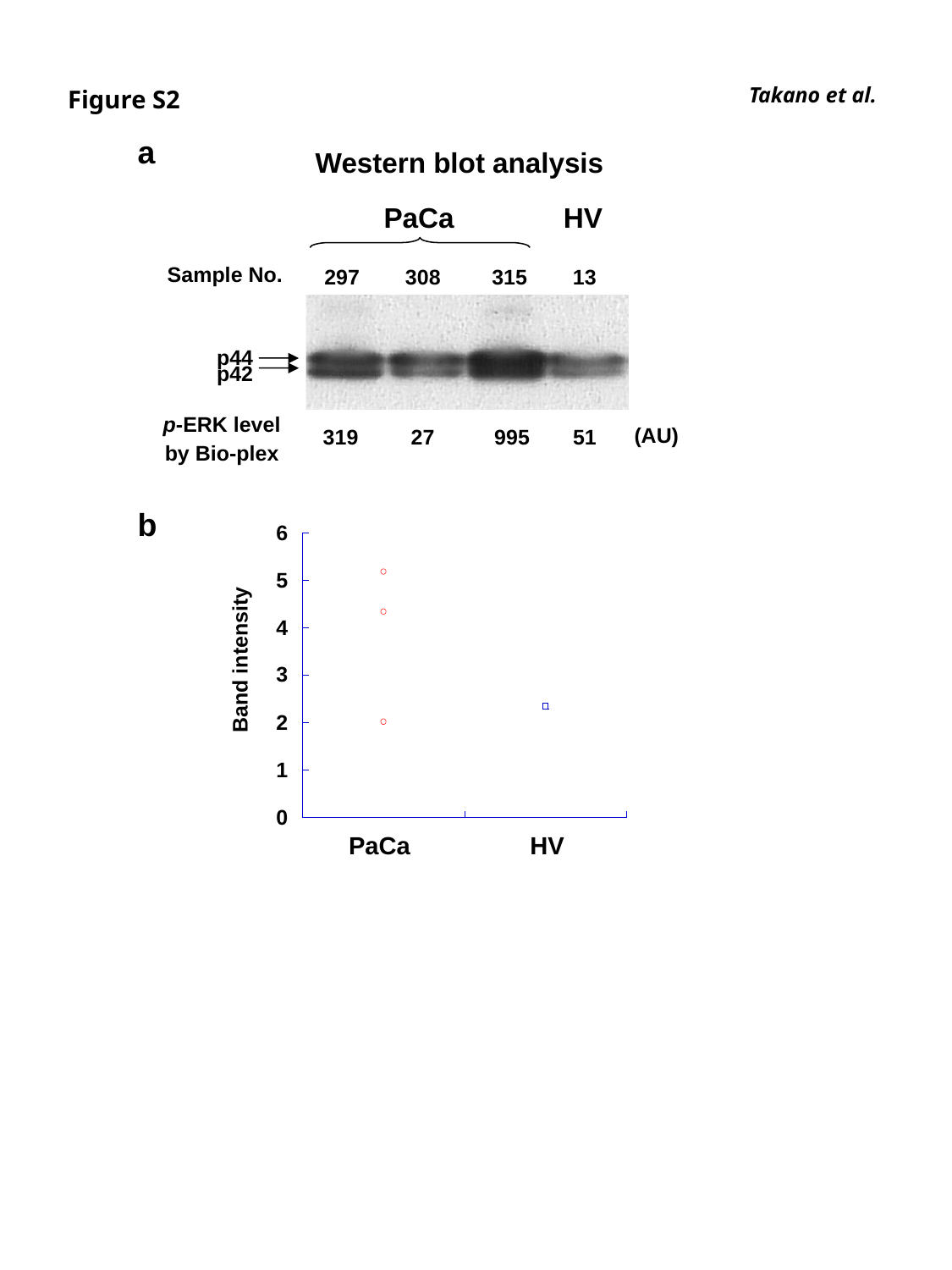

Takano et al.
Figure S2
a
Western blot analysis
PaCa
HV
Sample No.
297
308
315
13
p44
p42
p-ERK level
by Bio-plex
(AU)
27
995
51
319
b
6
5
4
Band intensity
3
2
1
0
HV
PaCa
