# Supplementary material for: Increased circulating cell signalling phosphoproteins in sera are useful for the detection of pancreatic cancer
Source: Br J Cancer. 2010 Jun 15;103(2):223–31. doi: 10.1038/sj.bjc.6605734 (PMC2906731; doi:10.1038/sj.bjc.6605734)
Supplement: Supplementary Table S1 [file 6605734x3.doc]

Table S1. Interference Assay

|  | Concentration | *p*-ERK1/2 | | | *p*-MEK1 | | |
| --- | --- | --- | --- | --- | --- | --- | --- |
| Substances | Low ; High |  | Low | High |  | Low | High |
| Total bilirubin | 1.3 ; 10.5 mg/dL |  | 31.5 | 35.0 |  | 9.5 | 11.0 |
| Conjugate bilirubin | 0.9 ; 9.8 mg/dL |  | 25.0 | 28.5 |  | 10.5 | 9.0 |
| Hemoglobin | 0.2 ; 2.4 g/L |  | 25.0 | 21.0 |  | 9.5 | 8.0 |
| Chyle | 1410 ; 14100 FTU |  | 29.0 | 24.5 |  | 10.0 | 8.5 |
