# Supplementary material for: Increased circulating cell signalling phosphoproteins in sera are useful for the detection of pancreatic cancer
Source: Br J Cancer. 2010 Jun 15;103(2):223–31. doi: 10.1038/sj.bjc.6605734 (PMC2906731; doi:10.1038/sj.bjc.6605734)
Supplement: Supplementary Table S2-1 [file 6605734x4.pdf]

Type	Description	p-Akt (75)	p-ERK1/2 (38)	p-GSK-3a/b (18)	p-IkB-a (58)	p-JNK (34)
C1	EGF HEK293	520	21313	8990	187	399
C2	EGF Hela	258	23301	6330	268	432
C3	INF-alpha Hela	288	1575	5475	281	176
C4	M-phase Hela	179	2285	3457	378	189
C5	NGFb PC12	870	24126	11485	417	1169
C6	TNF-a Hela	159	10530	4349	6185	311
C7	UV- HEK293	640	9808	9926	233	7410
C8	Untreated Hela Ly	146	727	2267	220	85
X1	PCa1	57	79	540	120	21
X2	PCa2	56	32	1158	100	18
X3	PCa3	55	117	1163	175	15
X4	PCa4	62	267	1397	200	21
X5	PCa5	60	74	1094	142	15
X6	PCa6	56	65	916	123	19
X7	PCa7	52	48	492	133	21
X8	PCa8	182	116	763	146	85
X9	PCa9	56	71	218	78	19
X10	PCa10	59	37	471	92	15
X11	PCa11	62	159	1467	177	20
X12	PCa12	138	62	1126	106	19
X13	PCa13	48	68	810	130	15
X14	PCa14	58	31	174	74	14
X15	PCa15	51	103	1010	104	21
X16	PCa16	57	13	596	83	19
X17	PCa17	59	63	1148	120	18
X18	PCa18	57	158	950	147	20
X19	PCa19	47	157	387	88	34
X20	PCa20	55	266	973	186	19
X21	PCa21	53	97	641	131	20
X22	PCa22	51	224	719	80	17
X23	PCa23	62	58	270	142	14
X24	PCa24	89	89	1040	87	24
X25	PCa25	62	27	1041	70	15
X26	PCa26	51	41	851	85	15
X27	HV1	47	16	1164	93	28
X28	HV2	47	53	602	89	19
X29	HV3	50	47	1250	180	17
X30	HV4	50	25	1031	89	16
X31	HV5	44	46	771	120	20
X32	HV6	54	39	1272	97	18
X33	HV7	51	21	1046	54	18
X34	HV8	54	21	1042	123	16
X35	HV9	56	22	1129	128	15
X36	HV10	56	18	1223	104	15
X37	HV11	54	12	1079	67	19
X38	HV12	49	7	425	71	12
X39	HV13	49	12	271	74	18
X40	HV14	51	9	293	73	15
X41	HV15	110	13	672	67	15
X42	HV16	50	8	633	70	16
X43	HV17	45	9	669	74	14
X44	HV18	50	6	370	68	14
X45	HV19	52	52	332	70	19
X46	HV20	53	8	638	67	22
X47	HV21	53	11	300	63	15
X48	HV22	51	12	856	73	14
X49	HV23	47	9	231	73	15
X50	HV24	44	14	208	65	16
X51	HV25	44	15	567	63	18
