# Supplementary material for: Increased circulating cell signalling phosphoproteins in sera are useful for the detection of pancreatic cancer
Source: Br J Cancer. 2010 Jun 15;103(2):223–31. doi: 10.1038/sj.bjc.6605734 (PMC2906731; doi:10.1038/sj.bjc.6605734)
Supplement: Supplementary Table S2-2 [file 6605734x5.pdf]

Type	Description	p-p38 MAPK (36)	p-p70 S6 Kinase (55)	p-p90RSK (35)	p-STAT2 (32)
C1	EGF HEK293	277	3601	430	88
C2	EGF Hela	420	6414	525	159
C3	INF-alpha Hela	237	2467	334	17551
C4	M-phase Hela	809	13980	327	124
C5	NGFb PC12	1108	9065	3047	122
C6	TNF-a Hela	508	3694	321	93
C7	UV- HEK293	1623	9670	309	134
C8	Untreated Hela Ly	155	619	230	87
X1	PCa1	51	200	105	23
X2	PCa2	39	223	156	21
X3	PCa3	43	243	115	23
X4	PCa4	58	330	149	24
X5	PCa5	49	231	111	21
X6	PCa6	43	180	103	20
X7	PCa7	44	237	118	21
X8	PCa8	72	341	199	64
X9	PCa9	44	242	121	18
X10	PCa10	50	223	123	18
X11	PCa11	43	270	121	23
X12	PCa12	47	245	122	23
X13	PCa13	49	227	114	20
X14	PCa14	40	115	73	18
X15	PCa15	55	222	125	18
X16	PCa16	39	162	86	19
X17	PCa17	50	241	115	20
X18	PCa18	49	288	137	21
X19	PCa19	46	463	109	17
X20	PCa20	57	269	142	20
X21	PCa21	56	500	216	18
X22	PCa22	68	217	110	22
X23	PCa23	56	207	109	21
X24	PCa24	93	1214	199	17
X25	PCa25	46	141	77	18
X26	PCa26	42	132	86	19
X27	HV1	50	185	115	18
X28	HV2	53	280	152	17
X29	HV3	51	218	93	19
X30	HV4	48	222	98	18
X31	HV5	50	213	127	18
X32	HV6	50	179	92	17
X33	HV7	55	164	85	13
X34	HV8	42	201	114	19
X35	HV9	46	172	97	19
X36	HV10	45	147	91	16
X37	HV11	47	174	86	15
X38	HV12	42	130	76	19
X39	HV13	37	118	63	16
X40	HV14	36	127	69	18
X41	HV15	44	179	70	16
X42	HV16	38	136	74	16
X43	HV17	35	101	60	17
X44	HV18	30	91	58	16
X45	HV19	39	125	63	16
X46	HV20	38	181	96	18
X47	HV21	42	131	86	14
X48	HV22	41	149	73	16
X49	HV23	38	115	71	16
X50	HV24	40	156	79	16
X51	HV25	39	342	151	18
