# Supplementary material for: Increased circulating cell signalling phosphoproteins in sera are useful for the detection of pancreatic cancer
Source: Br J Cancer. 2010 Jun 15;103(2):223–31. doi: 10.1038/sj.bjc.6605734 (PMC2906731; doi:10.1038/sj.bjc.6605734)
Supplement: Supplementary Table S2-3 [file 6605734x6.pdf]

Type	Description	p-STAT3 (52)	p-TrkA (73)	c-Jun (56)	CREB (19)	Histone H3 (77)
C1	EGF HEK293	43	124	2239	3251	280
C2	EGF Hela	290	184	1838	1220	587
C3	INF-alpha Hela	15400	134	299	803	509
C4	M-phase Hela	117	160	11518	3383	4296
C5	NGFb PC12	93	813	14366	2269	579
C6	TNF-a Hela	105	132	765	727	440
C7	UV- HEK293	59	142	21399	2481	284
C8	Untreated Hela Ly	176	123	109	246	151
X1	PCa1	19	70	90	129	41
X2	PCa2	22	68	52	110	47
X3	PCa3	23	69	30	113	47
X4	PCa4	22	75	32	84	48
X5	PCa5	20	67	27	107	46
X6	PCa6	19	64	36	72	38
X7	PCa7	22	65	47	75	50
X8	PCa8	36	122	77	157	86
X9	PCa9	22	71	26	136	45
X10	PCa10	24	67	27	96	45
X11	PCa11	20	73	27	101	51
X12	PCa12	21	88	30	110	59
X13	PCa13	20	73	21	67	42
X14	PCa14	20	55	18	42	39
X15	PCa15	21	68	32	108	48
X16	PCa16	18	61	39	41	34
X17	PCa17	25	67	30	81	39
X18	PCa18	21	73	30	199	45
X19	PCa19	25	113	34	143	154
X20	PCa20	21	65	29	138	53
X21	PCa21	22	177	31	146	126
X22	PCa22	31	69	33	118	60
X23	PCa23	64	75	123	197	45
X24	PCa24	30	370	126	101	303
X25	PCa25	20	57	27	43	37
X26	PCa26	21	62	32	48	38
X27	HV1	21	86	25	32	56
X28	HV2	26	106	37	61	78
X29	HV3	20	62	23	123	43
X30	HV4	23	94	26	47	56
X31	HV5	21	74	23	51	50
X32	HV6	19	70	844	827	39
X33	HV7	19	54	22	103	34
X34	HV8	23	64	38	84	48
X35	HV9	20	66	41	64	46
X36	HV10	20	57	80	36	41
X37	HV11	18	65	21	30	37
X38	HV12	20	54	23	25	40
X39	HV13	18	52	20	32	37
X40	HV14	18	51	53	54	37
X41	HV15	29	62	20	31	46
X42	HV16	20	60	23	34	50
X43	HV17	16	55	22	46	33
X44	HV18	19	54	21	24	31
X45	HV19	19	55	23	39	37
X46	HV20	18	80	51	19	55
X47	HV21	19	62	59	34	39
X48	HV22	19	57	48	47	36
X49	HV23	20	53	24	22	40
X50	HV24	19	51	25	24	42
X51	HV25	20	124	42	32	104
