# Supplementary material for: Increased circulating cell signalling phosphoproteins in sera are useful for the detection of pancreatic cancer
Source: Br J Cancer. 2010 Jun 15;103(2):223–31. doi: 10.1038/sj.bjc.6605734 (PMC2906731; doi:10.1038/sj.bjc.6605734)
Supplement: Supplementary Table S2-4 [file 6605734x7.pdf]

Type	Description	HSP27 (51)	IRS-1 (76)	MEK1 (40)	p53 (53)
C1	EGF HEK293	128	534	6072	781
C2	EGF Hela	6815	583	12571	137
C3	INF-alpha Hela	805	248	819	84
C4	M-phase Hela	7559	2028	975	68
C5	NGFb PC12	354	3040	6228	225
C6	TNF-a Hela	12333	192	2554	82
C7	UV- HEK293	194	847	1040	2394
C8	Untreated Hela Ly	1621	110	290	59
X1	PCa1	46	43	71	29
X2	PCa2	56	42	38	29
X3	PCa3	35	43	21	25
X4	PCa4	48	49	133	30
X5	PCa5	85	44	50	28
X6	PCa6	47	44	54	31
X7	PCa7	48	41	49	29
X8	PCa8	77	152	367	35
X9	PCa9	78	41	93	25
X10	PCa10	56	40	40	31
X11	PCa11	53	46	116	37
X12	PCa12	77	44	60	30
X13	PCa13	44	41	22	30
X14	PCa14	32	39	27	28
X15	PCa15	80	40	82	29
X16	PCa16	38	38	25	47
X17	PCa17	129	42	57	26
X18	PCa18	60	45	94	30
X19	PCa19	216	43	102	23
X20	PCa20	251	46	151	29
X21	PCa21	165	94	103	26
X22	PCa22	57	49	71	29
X23	PCa23	85	41	37	33
X24	PCa24	358	48	44	50
X25	PCa25	29	39	25	32
X26	PCa26	34	40	37	28
X27	HV1	120	42	93	25
X28	HV2	190	51	79	25
X29	HV3	67	39	52	29
X30	HV4	110	46	36	27
X31	HV5	59	41	88	30
X32	HV6	40	40	93	25
X33	HV7	29	36	37	27
X34	HV8	76	41	36	30
X35	HV9	67	42	45	28
X36	HV10	40	40	34	24
X37	HV11	54	38	25	24
X38	HV12	64	39	20	27
X39	HV13	38	37	26	24
X40	HV14	39	35	25	22
X41	HV15	107	37	19	31
X42	HV16	81	40	21	25
X43	HV17	29	36	18	26
X44	HV18	27	35	19	24
X45	HV19	34	35	36	21
X46	HV20	59	39	22	22
X47	HV21	27	36	20	25
X48	HV22	36	46	21	24
X49	HV23	43	32	22	27
X50	HV24	46	39	18	24
X51	HV25	76	41	26	27
