# Supplementary material for: Increased circulating cell signalling phosphoproteins in sera are useful for the detection of pancreatic cancer
Source: Br J Cancer. 2010 Jun 15;103(2):223–31. doi: 10.1038/sj.bjc.6605734 (PMC2906731; doi:10.1038/sj.bjc.6605734)
Supplement: Supplementary Figures and Tables Legends [file 6605734x8.doc]

**Supplementary data**

**Figure S1: The investigation of dynamic ranges for optimized phosphoprotein measurements**

For the determination of dynamic ranges for optimized measurement, serum samples were measured to assure two representative phosphoproteins in 4-point dilutions by Bio-Plex array.

Figure S1 shows that the curves of both *p*-ERK and *p*-MEK are fitted accurately with r^2^ > 0.98.

**Figure S2: Immunoprecipitation assay and Western blot analysis**

First, 10 mg of antibody-fixed affinity beads (Shiseido, Tokyo, Japan) was bound with 10 ug of rabbit monoclonal anti-phospho-ERK1/2 antibody (Cell Signaling Technology, MA, USA). Subsequently, 50 ul of each serum sample was mixed with 100 ug of the beads in 500 ul of 20 mM Tris-buffered saline at 4°C for 1 hour. After centrifugation (3000 *g*, at 4°C for 1 min), the pellets were eluted with 100 ul of 100 mM Glycine-HCl. After purification, the immunoadsorbed proteins were eluted with 50 µl SDS-PAGE sample buffer. The immunoprecipitated proteins were analyzed by Western blot as previously reported (Takano *et al*, 2008). Rabbit monoclonal anti-phospho-ERK1/2 antibody (Cell Signaling Technology, MA, USA; diluted 1:500) was used as a primary antibody, and goat anti-rabbit IgG HRP (MP Biomedicals Cappel, OH, USA; diluted 1:3000) was used as a secondary antibody.

Corresponding with Bio-Plex data, increased *p*-ERK1/2 expression was confirmed by Western blot analysis in sera from 3 PaCa patients and 1HV (Figure S2a). TotalLab TL120 software v2006 (Shimadzu Co., Kyoto, Japan) quantified the intensity of each protein band and the intensity was used as an index of the level of protein expression (FigureS2b).

**Table S1: Interference assay**

Interference assay was performed to evaluate whether any interference with the measurement of phosphoproteins (*p*-ERK1/2 and *p*-MEK1) by Bio-Plex was observed due to the presence of high levels of free bilirubin, conjugated bilirubin, hemolytic hemoglobin and chyle using Interference Check.A Plus (Sysmex Corporation, Kobe, Japan). The addition of these factors to control serum did not influence the level of *p*-ERK in serum (Table S1).

**Table S2: The measurement of 18 targeted cell signaling phosphoproteins levels in sera of the training set using the Bio-Plex^TM^ suspension array**
